# Systemic and local immunity following adoptive transfer of NY-ESO-1 SPEAR T cells in synovial sarcoma

**DOI:** 10.1186/s40425-019-0762-2

**Published:** 2019-10-24

**Authors:** Indu Ramachandran, Daniel E. Lowther, Rebecca Dryer-Minnerly, Ruoxi Wang, Svetlana Fayngerts, Daniel Nunez, Gareth Betts, Natalie Bath, Alex J. Tipping, Luca Melchiori, Jean-Marc Navenot, John Glod, Crystal L. Mackall, Sandra P. D’Angelo, Dejka M. Araujo, Warren A. Chow, George D. Demetri, Mihaela Druta, Brian A. Van Tine, Stephan A. Grupp, Albiruni R. Abdul Razak, Breelyn Wilky, Malini Iyengar, Trupti Trivedi, Erin Van Winkle, Karen Chagin, Rafael Amado, Gwendolyn K. Binder, Samik Basu

**Affiliations:** 1 Adaptimmune, Oxford, UK; 2 Adaptimmune, Philadelphia, PA USA; 3 National Cancer Institute, Bethesda, MD USA; 4 Stanford University School of Medicine, Stanford, CA USA; 5 Department of Medicine, Memorial Sloan Kettering Cancer Center, New York, NY USA; 6 Department of Sarcoma Medical Oncology, The University of Texas MD Anderson Cancer Center, Houston, TX USA; 7 City of Hope, Duarte, CA USA; 8 Dana-Farber/Harvard Cancer Center, Boston, MA USA; 9 Moffitt Cancer Center, Tampa, FL USA; 10 Washington University in St. Louis School of Medicine, St. Louis, MO USA; 11 Pediatric Oncology, The Children’s Hospital of Philadelphia, Philadelphia, PA USA; 12 Cancer Clinical Research Unit, Princess Margaret Cancer Centre, Toronto, ON Canada; 13 Sylvester Comprehensive Cancer Center, University of Miami, Miami, FL USA

**Keywords:** Adoptive immunotherapy, Synovial sarcoma, NY-ESO-1, Fludarabine, Cyclophosphamide, T cell, TCR, IL-15, Cytokine, Antigen loss, Checkpoint therapy, Engineered cell therapy

## Abstract

**Background:**

Gene-modified autologous T cells expressing NY-ESO-1^c259^, an affinity-enhanced T-cell receptor (TCR) reactive against the NY-ESO-1-specific HLA-A*02-restricted peptide SLLMWITQC (NY-ESO-1 SPEAR T-cells; GSK 794), have demonstrated clinical activity in patients with advanced synovial sarcoma (SS). The factors contributing to gene-modified T-cell expansion and the changes within the tumor microenvironment (TME) following T-cell infusion remain unclear. These studies address the immunological mechanisms of response and resistance in patients with SS treated with NY-ESO-1 SPEAR T-cells.

**Methods:**

Four cohorts were included to evaluate antigen expression and preconditioning on efficacy. Clinical responses were assessed by RECIST v1.1. Engineered T-cell persistence was determined by qPCR. Serum cytokines were evaluated by immunoassay. Transcriptomic analyses and immunohistochemistry were performed on tumor biopsies from patients before and after T-cell infusion. Gene-modified T-cells were detected within the TME via an RNAish assay.

**Results:**

Responses across cohorts were affected by preconditioning and intra-tumoral NY-ESO-1 expression. Of the 42 patients reported (data cut-off 4June2018), 1 patient had a complete response, 14 patients had partial responses, 24 patients had stable disease, and 3 patients had progressive disease. The magnitude of gene-modified T-cell expansion shortly after infusion was associated with response in patients with high intra-tumoral NY-ESO-1 expression. Patients receiving a fludarabine-containing conditioning regimen experienced increases in serum IL-7 and IL-15. Prior to infusion, the TME exhibited minimal leukocyte infiltration; CD163^+^ tumor-associated macrophages (TAMs) were the dominant population. Modest increases in intra-tumoral leukocytes (≤5%) were observed in a subset of subjects at approximately 8 weeks. Beyond 8 weeks post infusion, the TME was minimally infiltrated with a TAM-dominant leukocyte infiltrate. Tumor-associated antigens and antigen presentation did not significantly change within the tumor post-T-cell infusion. Finally, NY-ESO-1 SPEAR T cells trafficked to the TME and maintained cytotoxicity in a subset of patients.

**Conclusions:**

Our studies elucidate some factors that underpin response and resistance to NY-ESO-1 SPEAR T-cell therapy. From these data, we conclude that a lymphodepletion regimen containing high doses of fludarabine and cyclophosphamide is necessary for SPEAR T-cell persistence and efficacy. Furthermore, these data demonstrate that non-T-cell inflamed tumors, which are resistant to PD-1/PD-L1 inhibitors, can be treated with adoptive T-cell based immunotherapy.

**Trial registration:**

ClinicalTrials.gov, NCT01343043, Registered 27 April 2011.

## Introduction

Synovial sarcomas (SS) are tumors of mesenchymal origin that represent 5–10% of all soft tissue sarcomas. Most SS occur as a result of a translocation between the X chromosome and chromosome 18 resulting in SS18-SSX1, SS18-SSX2, and/or SS18-SSX4 fusion proteins [1]. Current therapeutic options for primary localized SS include surgical resection, radiotherapy, and chemotherapy. For patients with advanced or recurrent disease, chemotherapies and targeted therapies have limited efficacy. To date, no immunotherapies have been approved in SS, and clinical trials with checkpoint inhibitors have not shown durable benefit in this patient population [2–4].

PD-1/PD-L1 (programmed cell death) pathway inhibitors have shown durable clinical benefit in tumor histologies that exhibit T-cell infiltration, elevated levels of PD-L1 expression, and higher levels of nonsynonymous somatic mutation burden [5]. By comparison, SS are poorly infiltrated by T cells and have marginal PD-L1 expression [6, 7]. As in other translocation-driven tumors, SS also have a low overall mutational burden. This likely contributes to a low neo-antigen burden, which may at least partially explain the paucity of intra-tumoral T cells. Furthermore, SS exhibit low copy number alterations, which might additionally contribute to low tumor antigenicity [8]. One immunogenic antigen expressed in the majority (~ 70%) of SS tumors is the cancer-testis antigen NY-ESO-1 [9–11]. Studies with either an NY-ESO-1 vaccine or adoptively transferred NY-ESO-1-specific T cells alone or in combination with interleukin (IL)-2 have demonstrated that producing cell-mediated immune responses to NY-ESO-1 is a promising strategy in SS [12–14].

The results from the initial cohort of this pilot study testing genetically modified autologous T cells specific to the NY-ESO-1 peptide SLLMWITQC in patients with advanced metastatic SS have previously been published [14]. Patients were treated with T cells engineered to express an affinity-enhanced T-cell receptor (TCR) recognizing a human leukocyte antigen (HLA)-A*02-restricted NY-ESO-1/LAGE-1a-derived peptide (NY-ESO-1 SPEAR T cells; GSK 794) following lymphodepletion with cyclophosphamide and fludarabine [14]. In this cohort, we observed 50% response rate with a durable (30.9 weeks median duration of response) in patients with high intra-tumoral NY-ESO-1 expression. Long-term persistence and greater expansion of SPEAR T cells were seen in responding patients. Lastly, the persisting SPEAR T cells in the blood had a stem cell memory phenotype, were poly-functional, and did not express exhaustion markers [14]. Three additional cohorts were opened under this study to determine the impact of low antigen expression, reduced preconditioning, and the effects of fludarabine on safety and efficacy. Here we present the response data from these three additional cohorts, along with correlative data assessing the immunologic mechanisms of response and resistance in patients with advanced metastatic SS from all four cohorts of the pilot study.

## Material and methods

### Study design

The data and results presented are from a phase 1/2, nonrandomized, open-label study (NCT01343043). This study was conducted in compliance with the Declaration of Helsinki, and in accordance with local legal and regulatory requirements. Patients were recruited from 10 academic centers. The protocol was approved by each center’s Institutional Review Board, and informed consent forms were obtained for all patients.

The primary endpoint was overall response rate by response evaluation criteria in solid tumors (RECIST) version 1.1, defined as the proportion of patients with a confirmed complete response (CR) or partial response (PR). Additional endpoints were correlative studies to evaluate the persistence and phenotype of gene-marked T cells and to evaluate the serum and tumor biomarkers.

### Patients

Key eligibility criteria included patients aged 4 years or older with histologically confirmed SS that was unresectable, metastatic, progressive, persistent or recurrent (advanced disease), who were HLA-A*02 positive, and who had tumors that express the NY-ESO-1 tumor antigen. NY-ESO-1 expression varied across cohorts. Patients enrolled in cohorts 1, 3, or 4 had ≥50% of tumor cells express antigen at 2+ or 3+ staining by centralized immunohistochemistry (IHC). Patients enrolled in cohort 2 had lower expression: ≥1% of tumor cells expressing antigen at 1+ staining by IHC, but not ≥50% of tumor cells expressing 2+ or 3+ by IHC. The lymphodepletion regimen also differed across cohorts. Patients in cohorts 1 and 2 received fludarabine 30 mg/m^2^/day × 4 days and cyclophosphamide 1800 mg/m^2^/day × 2 days. Patients in cohort 3 received cyclophosphamide alone at 1800 mg/m^2^/day × 2 days, while patients in cohort 4 received a lower dose regimen of fludarabine 30 mg/m^2^/day × 3 days and cyclophosphamide 600 mg/m^2^/day × 3 days (Table 1). The transduced cell doses were similar across each cohort; the median cell dose overall was 2.67 × 10^9^ transduced T-cells. Although various HLA-A*02 alleles bind the target peptide and are then recognized by the NY-ESO-1^c259^TCR, the affinity of this binding varies. Patients in this study had HLA-A*02:01 or HLA-A*02:06 alleles, which have similar binding affinities. Patients must have previously received at least a doxorubicin and/or ifosfamide-containing regimen and have measurable disease according to RECIST v1.1. Patients were Eastern Cooperative Oncology Group (ECOG) performance status 0–1, or for children ≤10 years of age Lansky ≥60, had a life expectancy of > 3 months, and had a left ventricular ejection fraction of ≥40%. Laboratory assessments for eligibility were as follows: absolute neutrophil count ≥1000/mm^3^, platelet count ≥75,000/mm^3^, serum bilirubin < 2 mg/dl, alanine aminotransferase and aspartate aminotransferase ≤2.5 × upper limit of normal, and creatinine clearance of ≥60 ml/min. HLA typing by high-resolution testing was performed at a local laboratory or centrally at the American Red Cross (Philadelphia, PA). NY-ESO-1 testing was performed via IHC at a Clinical Laboratory Improvement Amendments certified pathology laboratory at the National Cancer Institute (Bethesda, MD) or at QualTek Labs (Goleta, CA). Disease response was classified according to RECIST v1.1, and radiological disease assessments were performed at weeks 4, 8, 12 and every 3 months thereafter. Patients who progressed are followed for long-term toxicity until death or for 15 years post infusion.

**Table 1:** Patients were treated in four cohorts

Cohort	Antigen expression by IHC	Lymphodepletion regimen
1	2+ or 3+ in ≥50% of tumor cells	Flu 30 mg/m^2^/d × 4 + Cy 1800 mg/m^2^/d × 2
2	> 1+ in > 1% but < 2+ or 3+ in ≥50% of tumor cells	Flu 30 mg/m^2^/d × 4 + Cy 1800 mg/m^2^/d × 2
3	2+ or 3+ in ≥50% of tumor cells	Cy 1800 mg/m^2^/d × 2
4	2+ or 3+ in ≥50% of tumor cells	Flu 30 mg/m^2^/d × 3 + Cy 600 mg/m^2^/d × 3

### Cell and vector manufacturing

Cells and vector were manufactured as previously described [15].

#### Cell manufacturing

Briefly, engineered T cells were manufactured at the Cell and Vaccine Production Facility at the University of Pennsylvania (Philadelphia, PA) for patients 201 and 202. The remaining patients’ cells were manufactured at Hitachi Chemical Advanced Therapeutics Solutions LLC (Allendale, NJ). Engineered T cells were generated from CD3^+^ T cells that were activated and expanded using αCD3/αCD28 antibody-conjugated beads (Life Technologies). T cells were transduced at a target multiplicity of infection of 1 transducing unit per cell.

#### Vector manufacturing

Briefly, the lentiviral vector is a self-inactivating vector derived from HIV-1. An EF1α promoter drives transgene expression. Vector was produced at the City of Hope (Duarte, CA) using transient transfection with four plasmids expressing the transfer vector, rev, VSV-G, and gag/pol, in 293 T cells. Supernatant was collected at multiple timepoints, clarified, treated with Benzonase® and concentrated by tangential flow filtration and centrifugation. Transduction potency was measured on primary human T cells.

### Assays for gene-modified T-cell persistence and phenotypic analysis

Research sample collection and initial processing were performed at clinical sites and then transferred to Cambridge Biomedical (Boston, MA), a commercial laboratory operating in compliance with Good Laboratory Practices, as described previously [14], for further processing and quantitative polymerase chain reaction (qPCR) analyses specific for vector sequences.

#### qPCR analyses

qPCR and subsequent analyses were performed as previously described [14].

#### Flow cytometry detection reagents

The following antibodies were used: cluster of differentiation (CD)4 BV605, CD8 BV650, CCR7 PE-CF594 (Becton Dickinson), CD45RA PECy7, CD28 BV421, CD127 BV711, PD-1 PerCP-Cy5.5, CD95 APC, CD62L BV785 (BioLegend, Dedham, MA), CD3 AF700, CD45RO FITC, CD27 APC-ef780 (eBioscience, San Diego, CA). The dead cell exclusion stain (Live/Dead Aqua) was purchased from Invitrogen (Carlsbad, CA). To detect NY-ESO-1^c259^TCR-expressing cells, purified anti-phycoerythrin-conjugated dextramer reagents specific for the HLA-A*02:01 SLLMWITQC complex (Immudex) were used at the manufacturer’s recommended concentrations.

#### T-cell cytotoxicity assay

The T-cell cytotoxicity assay was performed as previously described [14], with 5000 A375 cells plated per well. Due to limiting cell numbers, only a singlet was possible to test for the dextramer-positive population compared to duplicate wells for the dextramer-negative population.

### Multiplex cytokine analysis

Serum cytokine levels were quantified using Meso Scale Discovery (MSD; Rockville, MD) electro-chemiluminescence multiplex platform at Cambridge Biomedical. Human serum samples were blinded and tested in duplicate using the V-PLEX Pro inflammatory panel. An independent validation of V-PLEX was conducted by Cambridge Biomedical. Serum samples were tested according to the manufacturer recommended 1:2 dilution. V-PLEX assays were performed following the MSD recommended protocol.

### Assays of tumor biopsies

#### Transcriptomic analyses

RNA extractions and transcriptomics analyses were performed at HistoGeneX (Antwerp, Belgium). Tumor tissue was macro-dissected from 5 μm sections of FFPE tissues and total RNA was extracted using the High Pure RNA FFPE Micro Kit (Roche, Mannheim, Germany), following the manufacturer’s protocol. RNA was quantified using the Ribogreen kit (high range; Molecular Probes Inc.; Eugene, OR) on a Fluoroskan Ascent instrument (ThermoFisher Scientific; Waltham, MA). Seven standard dilution points ranging from 20 ng/mL to 1000 ng/mL were used to quantify samples across a wide range of concentrations. Sample purity was evaluated by measuring 260/280 nm and 260/230 nm ratios using a Nanodrop™ spectrophotometer (ThermoFisher). All samples were titrated to 14 ng/μL, and 100 ng total sample RNA was analyzed on the NanoString nCounter® system (NanoString; Seattle, WA), which consist of the nCounter® Prep Station and nCounter® Digital Analyzer (NanoString), using a commercially available pre-defined probe set (nCounter PanCancer Immune Profiling Panel, NanoString). Data normalization was done using the nSolver® Analysis Software (NanoString) [16]. Data were analyzed via nSolver software and R v3.3.1 (R Foundation for Statistical Computing) [17].

#### IHC

IHC was performed as previously described at either at a CLIA-certified clinical laboratory (QualTek Labs) or a CLIA-certified and Belgian Accreditation Organization and College of American Pathologists–accredited laboratory (HistoGeneX) [14].

#### Bright field imaging

Bright field imaging assays were performed as previously described [14].

#### RNA in situ hybridization

RNA in situ hybridization (RNAish) for Hs-TCR-Adapt-C1/Hs-CD3-C2 (duplex) was performed on automation platform using the RNAscope®2.5 LS Red kit and Duplex Reagent Kit (Advanced Cell Diagnostics, Inc.; Newark, CA) according to the manufacturer’s instructions. Briefly, 5 μm FFPE tissue sections were pretreated with heat and protease prior to hybridization with the target oligo probes. Preamplifier, amplifier, and horseradish peroxidase/alkaline phosphatase-labeled oligos were then hybridized sequentially, followed by chromogenic precipitate development. Each sample was quality controlled for RNA integrity with an RNAscope® probe specific to PPIB/POLR2A RNA and for background with a probe specific to bacterial dapB RNA. Specific RNA staining signal was identified as green (C1) and red (C2) punctate dots for duplex assay. Samples were counterstained with hematoxylin.

## Results

### NY-ESO-1 SPEAR T cells mediate tumor regression over several months across all cohorts

**Fig. 1:**
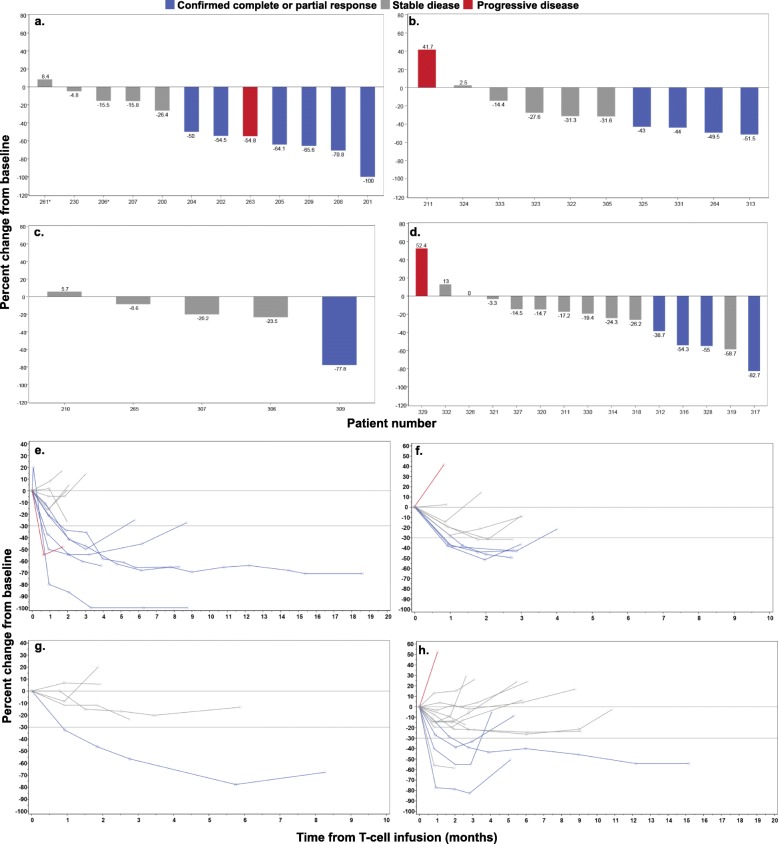
Clinical outcome in SS patients following NY-ESO-1 SPEAR T-cell infusion. Comparison of maximal tumor regression curves (waterfall plot) in 42 patients treated with NY-ESO-1 SPEAR T cells across four cohorts: **a** cohort 1, **b** cohort 2, **c** cohort 3, **d** cohort 4. Spider plots of tumor burden changes following NY-ESO-1 SPEAR T-cell infusion in 42 patients across four cohorts: **e** cohort 1, **f** cohort 2, **g** cohort 3, **h** cohort 4

Patients were grouped into four cohorts with varying levels of NY-ESO-1 antigen expression by tumors or pre-infusion lymphodepletion chemotherapy regimens (Table 1). We report 42 patients (data cut-off 4June18) who received a lymphodepleting chemotherapy regimen containing cyclophosphamide with or without fludarabine, followed by cell infusion on day 0. Patient response data for cohort 1 was previously reported [14], with the best overall responses (BOR) of stable disease (SD) in five patients, progressive disease (PD) in one patient, PR in 5 patients, and CR in one patient (Fig. 1a, b). Extended responses were seen, with a median duration of response (DOR) of 30.9 weeks (range 13.6–72.1 weeks). In cohort 2, which consisted of patients with low antigen expression, the BOR was SD in five patients, PD in one patient, and PR in four patients (Fig. 1). Median DOR was 10 weeks (range 7.9–12.9 weeks). Cohort 3 explored the efficacy of lymphodepletion with single agent cyclophosphamide. One out of five patients treated achieved a PR lasting 32 weeks, with SD as the BOR of the remaining four patients (Fig. 1). These results met protocol-defined futility, and this cohort was closed to further enrollment. Cohort 4 was subsequently opened to investigate lower doses of cyclophosphamide and fludarabine as compared with cohort 1. In this cohort, the BOR was SD for 10 patients, PD for one patient, and PR for four patients (Fig. 1). Median DOR was 16.3 weeks (range 14.1–54.0). All PRs were confirmed responses by RECIST v1.1. Although confirmed responses were seen in all four cohorts studied, the kinetics and depth of the responses varied, as shown by the spider plots for the four cohorts (Fig. 1e-h).

### SPEAR T-cell engraftment associated with pre-infusion lymphodepletion chemotherapy

**Fig. 2:**
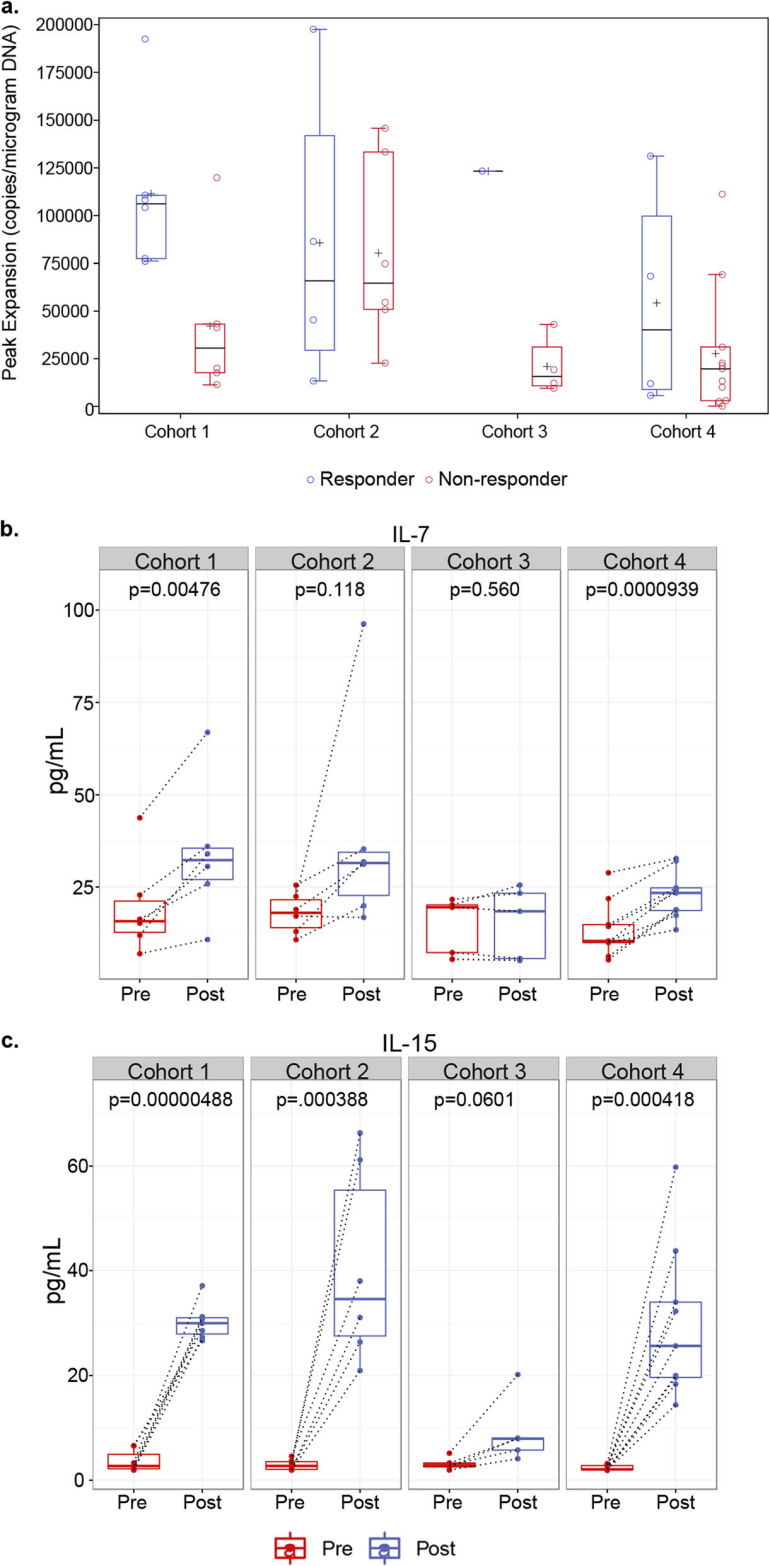
Pre-conditioning lymphodepletion regimen influences NY-ESO-1 SPEAR T-cell engraftment. **a** Peak expansion of transduced T cells in non-responders versus responders across all four cohorts was determined by measuring peak vector copies/μg of DNA in 42 patients treated with NY-ESO-1 SPEAR T cells. **b** IL-7 and **c** IL-15 levels in serum samples from 40 patients across all four cohorts were evaluated prior to (Pre-) and following (Post-) administration of pre-conditioning therapy, but prior to T-cell infusion. Box plots depict median, first and third quartiles. Dotted lines connect Pre- and Post- samples from the same patient. *p*-values between pre- and post-lymphodepletion in paired specimen in each cohort were calculated by the Wilcoxon matched-pairs signed-rank test

PCR was used to quantitate NY-ESO-1^c259^ vector copies following T-cell infusion. The peak levels reached for each patient were taken to represent the peak expansion of SPEAR T cells post infusion (Fig. 2a). As shown previously [14], peak levels were usually observed within the first 10 days post infusion and were significantly higher (*p* = 0.0411) in responders (median 106,174 vector copies/μg of genomic DNA; range 76,185–192,445) compared with non-responders (median 30,601 vector copies/μg of genomic DNA; range 11,265–119,883) in cohort 1 of this study (Fig. 2a). The median peak persistence for responders (median 65,875 vector copies/μg of genomic DNA; range 13,365–197,456) and non-responders (median 64,712 vector copies/μg of genomic DNA; range 22,627–145,791) in cohort 2 were nearly identical (Fig. 2a). The one responder in cohort 3 had higher peak persistence (123,314 vector copies/μg of genomic DNA) as compared with the median of non-responders (median 15,688 vector copies/μg of genomic DNA; range 9453–43,015) from this cohort (Fig. 2a). Responders in cohort 4 (median 40,137 vector copies/μg of genomic DNA; range 5677–131,176) had a slightly higher median peak persistence as compared with non-responders (median 19,650 vector copies/μg of genomic DNA; range 164–111,260; Fig. 2a). However, this difference was not statistically significant. Finally, transduced T cells were detected in all patients post-infusion, irrespective of responder status. These persistence data suggest that the overall dose of fludarabine and cyclophosphamide used to lymphodeplete patients, as well as the use of fludarabine and antigen expression, may impact the magnitude of SPEAR T-cell engraftment, response rate and durability.

To better elucidate the contributions of cyclophosphamide and fludarabine with peak persistence, we measured T-cell homeostatic cytokines prior to and immediately after lymphodepletion. We observed significant increases in serum IL-7 (Fig. 2b, Additional file 2: Figure S1a) and IL-15 (Fig. 2c, Additional file 2: Figure S1b) post lymphodepletion in patients who received both fludarabine and cyclophosphamide as a part of their pre-conditioning regimen (cohorts 1, 2, and 4). Patients receiving only cyclophosphamide as a part of their pre-conditioning regimen (cohort 3) did not have any significant changes in serum IL-7 following lymphodepletion (Fig. 2b). Furthermore, cohort 3 patients had substantially lower levels of IL-15 induction post-lymphodepletion as compared with patients from cohorts 1, 2, and 4 (Fig. 2c).

### Impact of SPEAR T-cell therapy on tumor microenvironment

**Fig. 3:**
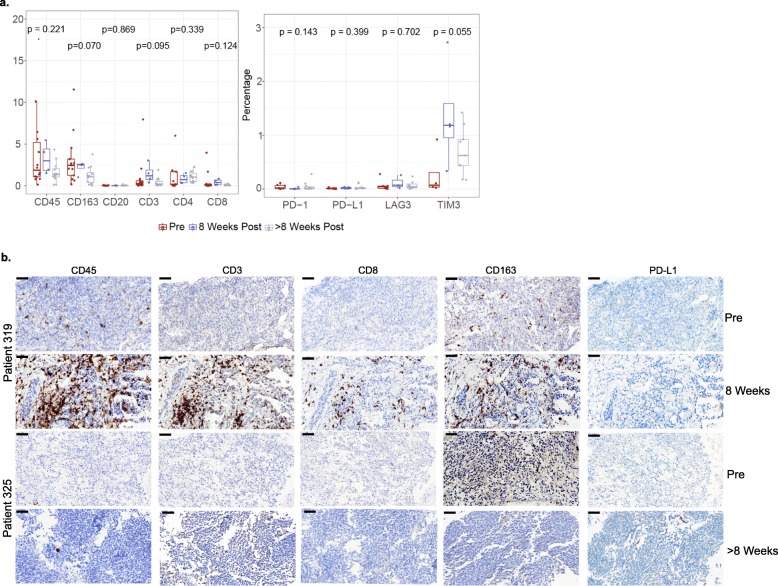
SPEAR T-cell therapy alters cellular infiltrate in tumor microenvironment. **a** Markers associated with immune cells and their function were evaluated at pre-infusion (red), and post- infusion at week 8 (blue) or beyond week 8 (grey) by IHC and plotted as a percentage of marker area within tumor area. Statistical significance in marker positivity between time points was determined by a two-way ANOVA test. **b** Immune marker expression in a representative region of pre- and post- infusion biopsies in one patient with increased leukocyte infiltration at week 8, and in another patient with minimal changes at >week 8 time point. Scale bar = 50 μm

SS exhibit minimal leukocyte infiltration [6, 7]. To investigate the tumor immune microenvironment in our patients, we characterized leukocyte infiltration in patient biopsies (Additional file 1: Table S1) taken prior to and following infusion by IHC staining (CD45, CD3, CD4, CD8, CD20, CD163, PD-L1, PD-1, TIM-3, and LAG-3). We also analyzed RNA extracted from pre- and post-infusion biopsies using a NanoString gene expression assay to assess a wider panel of immune-related genes. Prior to SPEAR T-cell infusion, there is minimal infiltration by CD3^+^ or CD8^+^ T cells in SS tumors and no detectable PD-L1 expression (Fig. 3a, b). The dominant leukocyte population in pre-infusion and post-infusion biopsies is CD163^+^ tumor-associated macrophages (TAM) (Fig. 3a, b).

To understand the impact of SPEAR T-cell therapy on the tumor microenvironment, we analyzed biopsies taken from patients either at 8 weeks or > 8 weeks post infusion. While there was not a significant difference in immune infiltration patterns across all biopsies after infusion, we did observe a trend towards an increased presence of CD3^+^ and CD8^+^ T cells at 8 weeks post infusion for a subset of patients, although the overall amount of infiltration remained quite low (≤5%) (Fig. 3a). While limited by the number of samples analyzed, there appeared to be heterogeneity in leukocyte infiltration at the 8-week post-infusion time point, even among responding patients. Patients 319 and 325 had target lesion shrinkages of 58.7 and 43%, respectively, at week 8 (Fig. 1b and d). Patient 319 from cohort 4 had an increase in CD45, CD3, CD8, and CD163 staining 8 weeks following SPEAR T-cell infiltration (Fig. 3b). This is in contrast to patient 325 from cohort 2, where there is no evidence of an increase in leukocyte infiltration at the 8-week time point (Fig. 3b).

### Tumor-associated antigen expression and antigen presentation are unaffected by SPEAR T-cell infusion

**Fig. 4:**
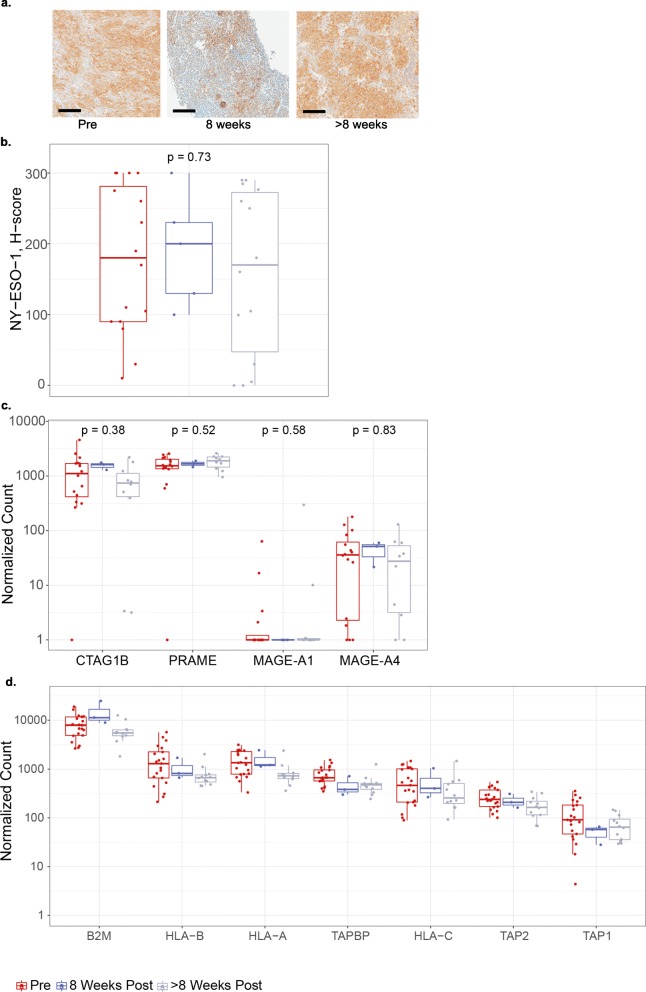
SPEAR T-cell therapy does not affect antigen expression or presentation. **a** Representative IHC images of NY-ESO-1 expression at each of the time points evaluated. Scale bar = 100 μm. **b** NY-ESO-1 protein expression H-scores as determined by IHC in pre- and post-infusion biopsies from all patients where a minimum of one post-infusion biopsy was evaluable (*N* = 15). Where > 1 biopsy per timepoint was evaluated, average H-score is shown. Mann-Whitney statistical test was used to evaluate changes between pre- and post-infusion time points. Tumor-associated antigen (**c**) and antigen-processing machinery (**d**). RNA expression shown as normalized counts as determined by the NanoString assay performed on pre- and post-infusion biopsies. Where more than one biopsy was collected and tested separately, points show the mean. Box-plots depict the median, along with first and third quartiles

We assessed NY-ESO-1 expression pre- and post-infusion to understand the interplay between target antigen expression and SPEAR T cell infusion (Fig. 4a, b). Across all patients, median NY-ESO-1 expression as detected by IHC did not change following T-cell infusion (Fig. 4b). There was no difference between median NY-ESO-1 expression in biopsies 8 weeks post infusion and NY-ESO-1 expression in biopsies > 8 weeks post infusion (Fig. 4a). Three patients appear to have low H-scores in their > 8 weeks biopsies. This observation may represent a limit of the H-score metric and reflects heterogeneity within the tumor, as comparable H-score values were observed in the baseline biopsies. Furthermore, in a subset of patients who progressed on therapy where biopsies were sampled prior to infusion and at progression, there was no change in median NY-ESO-1 expression (Additional file 3: Figure S2). We did not observe any changes in PRAME and MAGE-A4, other SS tumor-associated antigens [18] (Fig. 4c). Our results suggest NY-ESO-1 antigen loss is not associated with SPEAR T-cell infusion in SS and it does not appear to be a common mechanism of resistance in sarcoma.

The target for the NY-ESO-1 SPEAR TCR is a short amino acid sequence derived from NY-ESO-1 bound in the groove of HLA-A*02. The NY-ESO-1 peptide is generated by the proteasome, and successful loading of the peptide onto the HLA molecule requires specialized transport and chaperone proteins. Disruption of this process may render tumor cells resistant to TCR-targeted therapy. To further understand the relationship between antigen presentation and SPEAR T cell infusion, we assessed the mRNA expression of several genes implicated in antigen processing and presentation before and after T-cell infusion, and we did not detect any significant alterations in their expression that could be detected across all patients (Fig. 4d). While this analysis does not address mutations and/or loading of the NY-ESO-1 peptide, SLLMWITQC, onto HLA-A2 molecules, it suggests that SPEAR T-cell transfer did not impact these pathways.

### NY-ESO-1 SPEAR T cells can infiltrate tumors and maintain cytotoxic function long after infusion

**Fig. 5:**
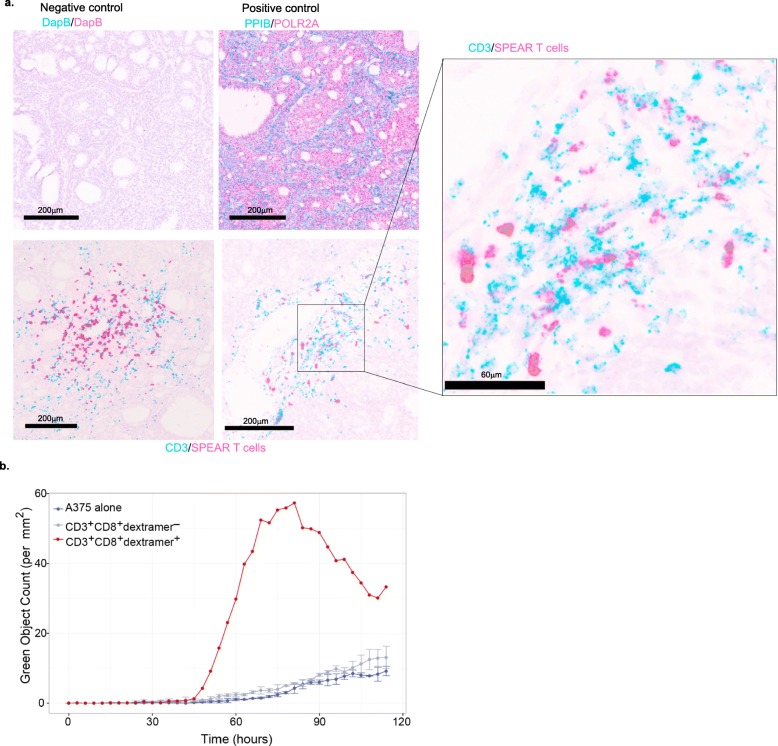
Adoptively transferred NY-ESO-1 SPEAR T cells maintain functionality long after infusion. **a** Representative fields for detection of negative control RNA (DapB), positive control RNA (PPIB, POLR2A), and CD3 or NY-ESO-1^c259^TCR RNA by RNAish in one patient’s tumor collected over 2 years post-infusion. **b** A375 target killing shown as green object count as determined by the Incucyte killing assay performed on flow sorted CD3^+^CD8^+^dextramer^+^ T cells (red line) and CD3^+^CD8^+^dextramer^−^ T cells (grey line) from a patient’s PBMCs collected 12 months post-infusion and on A375 alone (blue line)

Previous results have shown infiltration of T cells into SS tumors, but the ability of gene-modified T cells to traffic into the tumor microenvironment was unclear [7]. We developed an RNA-based probe specific to the mRNA of the NY-ESO-1^c259^ TCR to use in an RNAish assay. In a biopsy from patient 202 taken approximately 28 months following infusion, we detected NY-ESO-1-specific SPEAR T cells within the SS tumor microenvironment (Fig. 5a).

Although the ability of SPEAR T cells to traffic into tumors is necessary for anti-tumor activity, other aspects of T-cell functionality are needed to mount an effective anti-tumor response. We isolated circulating SPEAR T cells from the periphery of patient 316 approximately 12 months post infusion and assessed cytolytic capacity (Fig. 5b). Consistent with a prior report [14], SPEAR T cells maintained functionality 12 months post infusion.

## Discussion

Adoptive T-cell therapy using engineered receptors has shown great promise in the treatment of hematologic malignancies, as demonstrated by recent US Food and Drug Administration approvals for CD19-directed chimeric antigen receptors (CARs) in B-cell acute lymphoblastic leukemia (ALL) and in diffuse large B-cell lymphoma (DLBCL) [19–25]. Furthermore, efficacy data in multiple myeloma with B-cell maturation antigen-directed CARs [26] and NY-ESO-1 SPEAR T-cells [15] is encouraging. To date, engineered cell therapy in non-hematopoietic solid tumors has not demonstrated benefit in significant numbers of patients. Solid tumors pose unique challenges to adoptive T-cell therapies as compared with hematopoietic malignancies. These include but are not limited to heterogeneous antigen expression, physiologic barriers to T-cell trafficking, and an immunosuppressive tumor microenvironment [27]. SS is an ideal tumor target, since the fundamental oncogenic mechanisms of this translocation-related sarcoma drive aberrant expression of NY-ESO-1 through the defective SWI/SNF complex, which results in abnormal function of polycomb repressor complexes modulating gene expression [28]. Results from this study [14] (Fig. 1e-h) establish that some of these challenges can be overcome by NY-ESO-1 SPEAR T cells.

In this report, we expand upon findings reported previously [14] and include data from three additional cohorts. We continue to report overall meaningful clinical efficacy of NY-ESO-1 SPEAR T cells in SS of 36% in this treatment-resistant population, despite meeting futility and an early stop in the cohort that lacked fludarabine. Furthermore, we addressed the impact of pre-infusion lymphodepletion on outcome. Although many differing combinations of fludarabine and cyclophosphamide have been utilized in adoptive immunotherapy clinical trials with varying outcomes (reviewed in [29]), there is no consensus on the optimal lymphodepletion regimen for adoptive T-cell therapy, and much of the available data are from early phase trials testing CAR-T cells in hematologic malignancies [29–32]. The applicability of these findings to TCR-based adoptive immunotherapies in carcinomas and sarcomas have yet to be determined. Our results indicate that a fludarabine-containing preparative regimen is needed to maximize the benefit of SPEAR T-cell therapy. Elevated levels of serum IL-7 and IL-15 following a fludarabine-containing lymphodepletion regimen appear to associate with the engraftment of adoptively transferred T cells in cohort 1. The role of IL-7 and IL-15 in contributing to CAR-T proliferation and/or engraftment has been described extensively in hematological malignancies [32]. Our findings suggest that a similar phenomenon exists in adoptive TCR-based immunotherapy in sarcoma. A preparative regimen consisting solely of cyclophosphamide is not sufficient for optimal SPEAR T-cell engraftment in the studied clinical setting, as evidenced by poor peak persistence in four out of the five patients treated in cohort 3. The only responder in this cohort was a pediatric patient who had the highest post-conditioning serum levels of IL-15. Persisting SPEAR T cells isolated from this patient, approximately 1 year after infusion, maintained ex vivo cytotoxicity, further suggesting that fludarabine itself is unlikely to be contributing to long-term anti-tumor activity (Fig. 5b). Cyclophosphamide does have a modest impact on IL-7 and IL-15 induction, but this induction appears to be sub-optimal for most patients when not used in conjunction with fludarabine. Based on our findings, we would strongly suggest a lymphodepletion regimen containing a high dose of fludarabine with cyclophosphamide for SS patients being treated with SPEAR T-cells.

We also investigated the contribution of pre-infusion antigen expression to the magnitude and duration of clinical response. Our results indicate that when given comparable lymphodepletion regimens, patients with higher intra-tumoral NY-ESO-1 expression (cohort 1) are more likely to have deeper and longer responses as compared with patients with lower NY-ESO-1 expression (cohort 2; Fig. 1). There was not an appreciable difference in peak persistence between responders and non-responders in cohort 2 (Fig. 2a), despite a preparative regimen incorporating fludarabine. This observation suggests that SPEAR T-cell engraftment is at least in part driven by antigen expression. As SPEAR T cells utilize an affinity-enhanced TCR, their proliferation should depend on the quantity of SLLMWITQC-HLA-A*2:01 and/or -HLA-A*2:06 complexes present within the tumor or in peripheral lymphoid tissue. This suggests a positive correlative relationship between overall NY-ESO-1 intra-tumoral expression and peptide-major histocompatibility complex (MHC) expression.

Patients with lower levels of pre-infusion intra-tumoral NY-ESO-1 expression still had meaningful responses (Fig. 1). Our data suggest that the differences in response rates between cohort 1 and cohort 2 are largely dependent upon target antigen expression (Fig. 1). As such, increasing NY-ESO-1 expression may be an attractive strategy to improve the magnitude and duration of response. DNA methyl transferase inhibitors (DNMTi) represent one such strategy, as they have been shown to induce cancer-testis antigen expression [33–35]. As of this publication, there is currently one open trial using engineered T cells specific to an NY-ESO-1 peptide being used in conjunction with decitabine (NCT03017131) in ovarian cancer. Additional studies will be needed to understand the contribution of intra-tumoral target antigen and response in solid malignancies.

NY-ESO-1 is an immunogenic intracellular cancer-testis antigen expressed across a variety of tumors but absent in non-malignant tissue [36]. This makes it an attractive target for TCR-based therapies, and consequently, numerous immune therapies targeting NY-ESO-1 are in clinical development [36]. A unique mechanism of resistance to adoptive cell therapy is immune escape via loss of target antigen. Antigen loss has been described with CARs in the setting of hematologic malignancies [15, 37, 38]. However, antigen loss has not been widely described in non-hematopoietic solid tumors [39]. To address this potential mechanism of resistance to our therapy, we biopsied tumors from patients enrolled on our study prior to and following infusion. Our data demonstrate that loss of NY-ESO-1 protein or mRNA in SS patients treated with SPEAR T cells is not a widespread phenomenon, possibly due to the aberrant epigenetic mechanisms in SS tumors caused by the defective SWI/SNF complex [28]. In fact several patients received second infusions after confirmation of antigen presence. Of 10 patients treated, one patient experienced a PR (duration of at least 4 weeks prior to data cut-off) and another patient had a CR (duration of at least 8 weeks prior to data cut-off). Following adoptive T-cell transfer, only three biopsies out of 20 biopsies tested had low antigen expression by IHC (Fig. 4b, Additional file 3: Figure S2). Additionally, the expression of other tumor-associated antigens PRAME and MAGE-A4 [6] remained stable across all time points, implying multiple targets are available for TCR-based therapies. Our data suggest that immune evasion via antigen loss is probably not a significant mechanism of resistance in SS patients treated with SPEAR T cells.

Loss of genes responsible for antigen presentation, including but not limited to MHC class I and β2-microglobulin, have been associated with disease progression during checkpoint inhibitor therapy in melanoma [40, 41]. To date, the prevalence of these mechanisms of resistance to checkpoint inhibition is not yet well characterized across other cancers [42, 43]. Although SPEAR T-cell therapy is distinct from checkpoint blockade, defects in or loss of antigen presentation and processing represent shared potential mechanisms of resistance. In addition to investigating target antigen expression from biopsies, we also explored these potential mechanisms of resistance. Our data indicate that transcriptional loss of expression of genes responsible for antigen processing and presentation are not implicated in progression on SPEAR T-cell therapy in SS. Loss of heterozygosity has recently been described as a significant mechanism of resistance for immunotherapy [42]. Specific loss or mutations of the HLA-A*02 allele or defects in SLLMWITQC peptide loading onto HLA-A*02 have not yet been ruled out as mechanisms of resistance in our study.

The ability of the adoptively transferred T cells to traffic to and subsequently function within the tumor microenvironment is critically important, as T cells are generally administered systemically and must traffic to metastatic disease sites. We analyzed biopsies for immune markers associated with response to and progression on immune therapy. In agreement with previous studies, pre-infusion biopsies from patients enrolled in this trial were minimally infiltrated with T cells and did not have appreciable PD-L1 expression [6, 7]. These data are consistent with the lack of efficacy observed to date with checkpoint inhibitors in SS [3]. Despite this challenge, our TCR-modified T cells were able to traffic into the tumor microenvironment in selected samples studied. These emerging data suggest that SPEAR T-cell therapy could be an attractive modality to target tumors that are poorly infiltrated by T cells, which are not suitable for other immune therapies, such as PD-1/PD-L1 blockade [27, 44].

Global leukocyte infiltration of the SS microenvironment is relatively low post infusion compared to infiltration observed in inflamed tumor types, where > 30% infiltration is often observed [45]. In addition to low amounts of infiltration, a relatively high abundance of CD163^+^ TAM was observed across all time points analyzed. These macrophages are associated with a tolerogenic M2 phenotype [46] and have been associated with poor outcomes in sarcoma and other tumors [6, 47]. Additional studies are needed to elucidate the relationship between CD163^+^ TAM and SPEAR T cells.

## Conclusions

We are encouraged by the clinical results from this study of adoptive T-cell therapy for SS using an affinity-enhanced NY-ESO-1^c259^ TCR. Analyses have revealed the contribution of intra-tumoral target antigen expression and fludarabine to T-cell engraftment. These data provide an initial rationale as to how tumors with histologies resistant to checkpoint blockade can be successfully targeted with adoptive T-cell therapy. Furthermore, these data are the first to demonstrate successful infiltration of solid tumors by SPEAR T cells, which are able to kill tumor cells. Our data also suggest that antigen loss or alterations in the expression of antigen processing proteins are not primary mechanisms of resistance. Moreover, the therapeutic efficacy may be enhanced through use of a high dose fludarabine-containing preparative lymphodepletion regimen, by promoting greater engraftment at the tumor site, and through modulation of TAM.

## Supplementary information

**Additional file 1: Table S1.** Patient biopsies.

**Additional file 2: Figure S1.** Pre-conditioning lymphodepletion regimen influences IL-7 and IL-15 production. Ratio of serum IL-7 (a) and IL-15 (b) plotted pre and post-lymphodepletion. Box plots depict mean, first and third quartiles. *p*-values between pre- and post-lymphodepletion in paired specimen in each cohort were calculated by the Wilcoxon matched-pairs signed-rank test.

**Additional file 3: Figure S2.** Change in antigen expression at progression. NY-ESO-1 protein expression H-scores as determined by IHC in pre-infusion and post-progression biopsies from all patients whose progression biopsies were evaluable (*N* = 15). Paired Mann-Whitney U statistical test was used to evaluate changes between pre-infusion and progression time points.

## Data Availability

The NY-ESO-1 program was transitioned from Adaptimmune to GlaxoSmithKline in July 2018. Information on GlaxoSmithKline’s data sharing commitments and access requests to anonymized individual participant data and associated documents can be found online (https://www.clinicalstudydatarequest.com/Default.aspx).

## Abbreviations

BOR: Best overall response
CAR: Chimeric antigen receptor
CR: Complete response
DOR: Duration of response
ECOG: Eastern Cooperative Oncology Group
HLA: Human leukocyte antigen
IHC: Immunohistochemistry
IL: Interleukin
MHC: Major histocompatibility complex
PBMC: Peripheral blood mononuclear cell
PD: Progressive disease
PD-L1: Programmed cell death ligand 1
PR: Partial response
qPCR: Quantitative polymerase chain reaction
RECIST: Response evaluation criteria in solid tumors
RNAish: RNA in situ hybridization
SPEAR: Specific peptide enhanced affinity receptor
SS: Synovial sarcoma
TAM: Tumor-associated macrophage
TCR: T-cell receptor
TME: Tumor microenvironment
