# Supplementary material for: Systemic and local immunity following adoptive transfer of NY-ESO-1 SPEAR T cells in synovial sarcoma
Source: J Immunother Cancer. 2019 Oct 24;7:276. doi: 10.1186/s40425-019-0762-2 (PMC6813983; doi:10.1186/s40425-019-0762-2)
Supplement: Supplementary file 1 — Additional file 1: Table S1. Patient biopsies. [file 40425_2019_762_MOESM1_ESM.docx]

**Table S1. Patient biopsies.**

| **Patient ID** | **Cohort** | **Responder (Y/N)** | **Pre** | **8 weeks post** | **> 8 weeks post** | **Figure** |
| --- | --- | --- | --- | --- | --- | --- |
| 211 | 2 | N | Y |  | Y | 3A |
|  |  |  | Y |  | Y | 4A |
|  |  |  | Y |  | Y* | S2 |
|  |  |  | Y |  | Y | 4C, 4D |
| 265 | 3 | N | Y | Y | Y | 3A |
|  |  |  | Y | Y | Y | 4A |
|  |  |  | Y | Y* | Y* | S2 |
|  |  |  | Y |  | Y | 4C, 4D |
|  |  |  | Y | Y | Y | 4B |
| 202 | 1 | Y | Y |  | Y | 3A |
|  |  |  | Y |  | Y | 4A |
|  |  |  | Y |  | Y* | S2 |
|  |  |  |  |  | Y | 4C, 4D |
| 209 | 1 | Y |  |  | Y | 3A |
|  |  |  | Y |  | Y | 4A |
|  |  |  | Y |  | Y* | S2 |
|  |  |  | Y |  |  | 4C, 4D |
| 306 | 3 | N | Y |  |  | 3A |
|  |  |  |  |  |  | 4A |
|  |  |  |  |  |  | S2 |
|  |  |  |  |  |  | 4C, 4D |
| 307 | 3 | N | Y |  |  | 3A |
|  |  |  |  |  |  | 4A |
|  |  |  |  |  |  | S2 |
|  |  |  | Y |  |  | 4C, 4D |
| 314 | 4 | N | Y |  |  | 3A |
|  |  |  | Y |  | Y | 4A |
|  |  |  | Y |  | Y* | S2 |
|  |  |  | Y |  |  | 4C, 4D |
| 322 | 2 | N | Y |  | Y | 3A |
|  |  |  | Y |  | Y | 4A |
|  |  |  | Y |  |  | S2 |
|  |  |  | Y |  | Y | 4C, 4D |
| 305 | 2 | N |  |  | Y | 3A |
|  |  |  | Y |  | Y | 4A |
|  |  |  | Y |  | Y* | S2 |
|  |  |  |  |  | Y | 4C, 4D |
| 321 | 4 | N | Y | Y | Y | 3A |
|  |  |  | Y | Y | Y | 4A |
|  |  |  | Y | N | Y* | S2 |
|  |  |  | Y | Y | Y | 4C, 4D |
| 313 | 2 | N |  |  | Y | 3A |
|  |  |  | Y |  | Y | 4A |
|  |  |  | Y |  | Y* | S2 |
|  |  |  |  |  | Y | 4C, 4D |
| 319 | 4 | N | Y | Y |  | 3A |
|  |  |  | Y | Y |  | 4A |
|  |  |  | Y | Y* |  | S2 |
|  |  |  | Y | Y |  | 4C, 4D |
|  |  |  | Y | Y |  | 3B |
| 325 | 2 | Y | Y |  | Y | 3A |
|  |  |  | Y | Y | Y | 4A |
|  |  |  | Y |  | Y* | S2 |
|  |  |  | Y |  | Y | 4C, 4D |
|  |  |  | Y |  | Y | 3B |
| 320 | 4 | N |  | Y |  | 3A |
|  |  |  | Y | Y |  | 4A |
|  |  |  |  |  |  | S2 |
|  |  |  | Y |  |  | 4C, 4D |
| 328 | 4 | Y | Y |  | Y | 3A |
|  |  |  | Y |  | Y | 4A |
|  |  |  | Y |  | Y* | S2 |
|  |  |  | Y |  | Y | 4C, 4D |
| 330 | 4 | N | Y |  |  | 3A |
|  |  |  |  |  |  | 4A |
|  |  |  |  |  |  | S2 |
|  |  |  | Y |  |  | 4C, 4D |
| 329 | 4 | N | Y |  |  | 3A |
|  |  |  |  |  |  | 4A |
|  |  |  |  |  |  | S2 |
|  |  |  | Y |  |  | 4C, 4D |
| 309 | 3 | Y |  |  | Y | 3A |
|  |  |  | Y |  | Y | 4A |
|  |  |  | Y |  | Y* | S2 |
|  |  |  |  |  | Y | 4C, 4D |
| 318 | 4 | N |  |  | Y | 3A |
|  |  |  | Y |  | Y | 4A |
|  |  |  | Y |  | Y* | S2 |
|  |  |  |  |  |  | 4C, 4D |
| 317 | 4 | Y |  |  |  | 3A |
|  |  |  | Y |  | Y | 4A |
|  |  |  | Y |  | Y* | S2 |
|  |  |  | Y |  |  | 4C, 4D |
| 320 | 4 | N |  |  |  | 3A |
|  |  |  | Y | Y |  | 4A |
|  |  |  |  |  |  | S2 |
|  |  |  |  | Y |  | 4C, 4D |
| 316 | 4 | Y |  |  |  | 3A |
|  |  |  |  |  |  | 4A |
|  |  |  |  |  |  | S2 |
|  |  |  | Y |  |  | 4C, 4D |

*Biopsies were collected at or after progression
