# Supplementary figures and images for: Systemic and local immunity following adoptive transfer of NY-ESO-1 SPEAR T cells in synovial sarcoma
Source: J Immunother Cancer. 2019 Oct 24;7:276. doi: 10.1186/s40425-019-0762-2 (PMC6813983; doi:10.1186/s40425-019-0762-2)

**a.**

IL-7

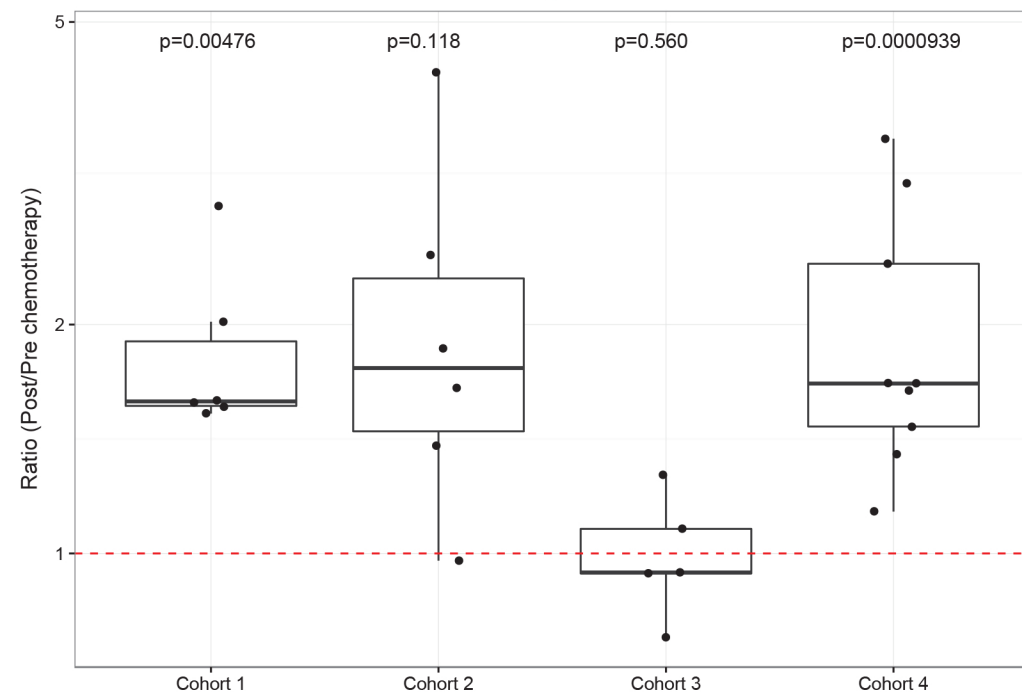**b.**

IL-15

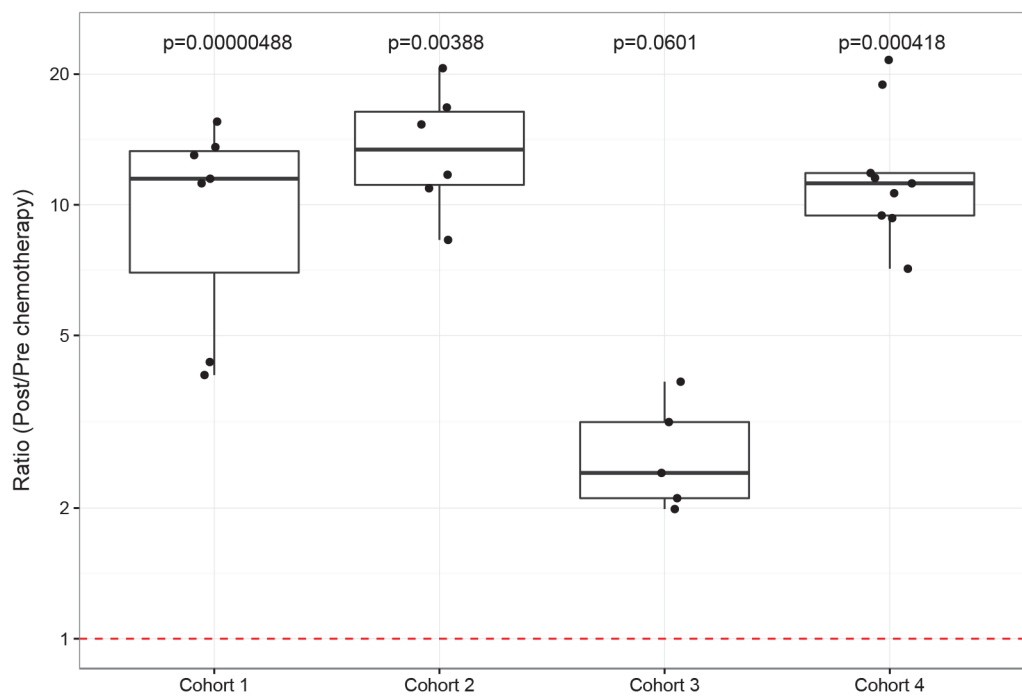

Supplement: Supplementary file 2 — Additional file 2: Figure S1. Pre-conditioning lymphodepletion regimen influences IL-7 and IL-15 production. Ratio of serum IL-7 (a) and IL-15 (b) plotted pre and post-lymphodepletion. Box plots depict mean, first and third quartiles. p-values between pre- and post-lymphodepletion in paired specimen in each cohort were calculated by the Wilcoxon matched-pairs signed-rank test. [file 40425_2019_762_MOESM2_ESM.pdf]

NY-ESO-1, H-score

$p = 0.32$

300

200

100

0

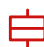

Pre-infusion

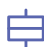

Progression

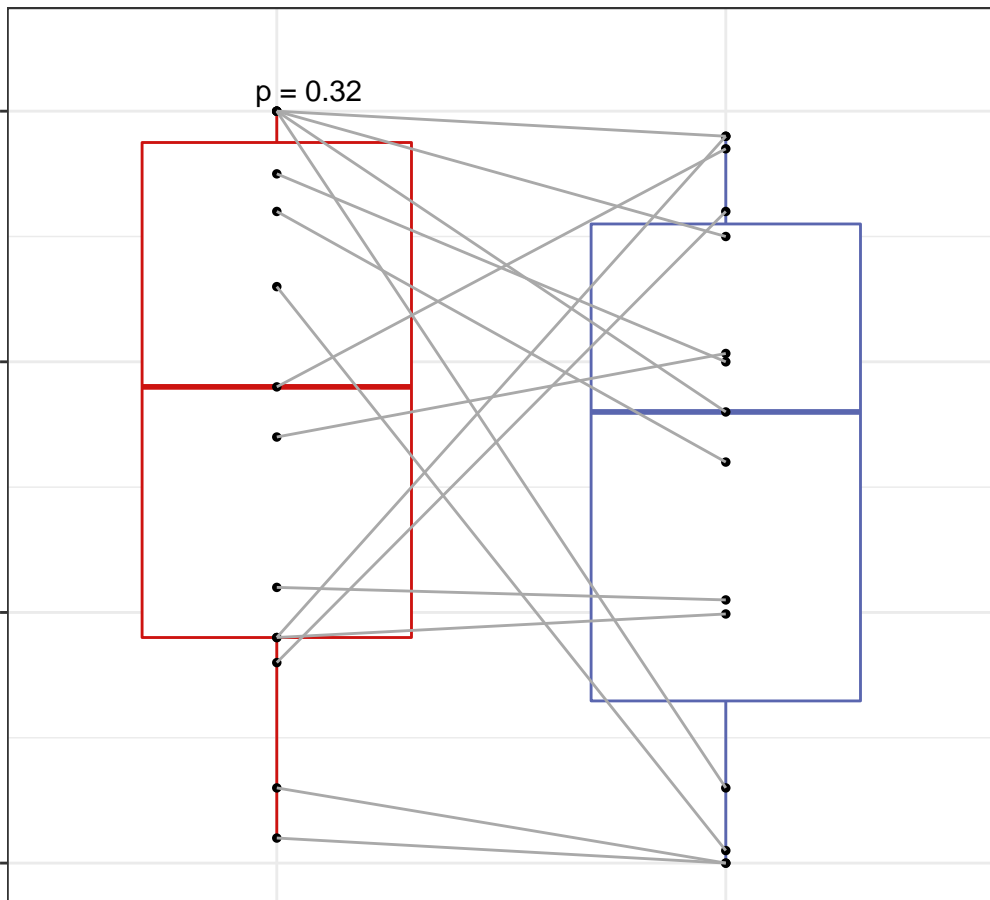

Supplement: Supplementary file 3 — Additional file 3: Figure S2. Change in antigen expression at progression. NY-ESO-1 protein expression H-scores as determined by IHC in pre-infusion and post-progression biopsies from all patients whose progression biopsies were evaluable (N = 15). Paired Mann-Whitney U statistical test was used to evaluate changes between pre-infusion and progression time points. [file 40425_2019_762_MOESM3_ESM.pdf]
